# Association between Adiponectin Concentrations and Cardiovascular Disease in Diabetic Patients: A Systematic Review and Meta-Analysis

**DOI:** 10.1371/journal.pone.0078485

**Published:** 2013-11-04

**Authors:** Zhenjie Wu, Yunjiu Cheng, Lynn Htet Htet Aung, Bixun Li

**Affiliations:** 1 Department of General Internal Medicine, Tumor Affiliated Hospital of Guangxi Medical University, Nanning, Guangxi, China; 2 Department of Cardiology, The First Affiliated Hospital, Sun Yat-Sen University, Guangzhou, Guangdong, China; 3 Department of Cardiology, Institute of Cardiovascular Diseases, The First Affiliated Hospital, Guangxi Medical University, Nanning, Guangxi, China; Kurume University School of Medicine, Japan

## Abstract

**Background:**

This systematic review and meta-analysis of prospective studies evaluates the association between adiponectin concentrations and risk of cardiovascular disease (CVD) in individuals with diabetes mellitus (DM).

**Methods:**

PubMed and Embase were searched for prospective studies on the association of adiponectin concentrations and risk of CVD up to June 2013. Random-effect model was selected to pool the relative risk (RR) and 95% CI.

**Results:**

Five prospective cohort studies and one nested case-control studies met the included criterion. The estimated summary RR and 95% CI of five prospective cohort studies for type 2 diabetes comparing top vs low tertile of adiponectin concentrations was 0.99 (95% CI: 0.67–1.45), with significant heterogeneity between studies (*p* = 0.037, *I* ^2^ = 60.9%). This heterogeneity was explained by one study conducted in Korean.

**Conclusions:**

This study represents the first meta-analysis between adiponectin levels and CVD in diabetic patients and indicated no association was found. This result should be verified further by large sample size, long duration of follow-up, and well-designed prospective clinical trials.

## Introduction

Adiponectin, a 244 amino acid collagen-like protein encoded by the AdipoQ gene in humans [1], is decreased in obesity [2], type 2 diabetes mellitus (DM), and those with coronary heart disease (CHD) [3], and thus have been hypothesized to have insulin sensitizing, anti-inflammatory and anti-atherogenic activities [4]. Meanwhile, atherosclerosis is one of the most common causes of cardiovascular disease, which remains the biggest cause of deaths in the world [5]. Most previous studies have accessed the association between adiponectin concentrations and risk of cardiovascular disease (CVD), and several meta-analyses reported positive [6] or inversed [7], [8] association or no statistically significant [6], . One reason for explaining these controversial results may be the pre-existing disease in study population [9], such as diabetics, patients on hemodialysis, and CHD patients. Diabetes can not only cause CVD, one of its long-term complications, but also doubles the risk of CVD [11]. So, it is interestingly to investigate the relationship between adiponectin concentration and the risk of CVD in diabetic patients. Rare data on the association between adiponectin concentration and risk of CVD in diabetic patients have been reported and the results are controversial [12]–[17]. We therefore conducted a systematic review and study-level meta-analysis of prospective epidemiological data to further evaluate this association in persons with type 1 or type 2 diabetes.

## Methods

### Search

The electronic databases MEDLINE and EMBASE (up to June 2013) were searched for relevant studies on the association between adiponectin (including total, high molecule weigh (HMW)) concentrations and risk of CVD in diabetic patients. For CVD, a complete description of the endpoint criteria must be provided, or referred to in previously published articles (CVD was defined as CHD, stroke, cardiac arrest, heart failure, peripheral artery disease, and sudden death; CHD was defined as acute myocardial infarction, angina pectoris, and other ischemic heart disease). The following terms were used for our search: 1) adiponectin, apM1, AdipoQ, Acrp30, GBP28, and C1q; 2) cardiovascular diseases, coronary disease, coronary thrombosis, myocardial ischemia, myocardial infarction, coronary stenosis, coronary restenosis, atherosclerosis, cerebrovascular disorders, stroke, and heart failure; 3) diabetes mellitus and diabetic; 4) prospective cohort studies and nested case-control studies. No language restriction was imposed. Reference lists from the identified articles were manually examined for relevant articles. We also check prior reviews to be sure that most relevant studies have been identified. The present analysis accorded with the preferred reporting items for systematic reviews and meta-analyses (PRISMA) guidelines (when appropriate) for a systematic review of prevalence (Checklist S1). [18]

### Study selection

Two independent reviewers identified studies meeting the following inclusion criteria: (i) the study evaluated the association between adiponectin concentrations and risk of CVD in patients with DM; (ii) the study design was a prospective cohort study or nested case-control study; (iii) the study provide or allow calculation of the relative risk (RR) with its corresponding 95% CI; (iv) end point included CVD; (v) the duration of follow-up was at least 1 year. Studies not meeting these criteria were excluded.

### Data extraction

Two reviewers independently extracted data using standardized data extraction forms. Discrepancies were resolved by group discussion. We extracted information from each publication: first author’s last name, year of publication, study location, study design (prospective cohort or nested case-control), study population (type 1 or type 2 diabetes mellitus), patients with preexisting CVD (%), number of participants and events, age (mean or median), type of adiponectin (total, HMW), duration of follow-up, assessments of outcomes (CVD, CHD, and stroke), results, measurements of risks (per standard deviation (SD) increase, tertile, quartile, and equal two groups), adjustment, and quality score. We assessed these nonrandomized studies’ quality using the Newcastle-Ottawa quality assessment scale, which evaluated studies’ quality in meta-analyses based on three items: patient selection, comparability of groups and ascertainment of outcome. [19] Studies were evaluated on an ordinal star scoring scale with higher scores on behalf of higher quality studies.

### Statistical analysis

We used multivariable-adjusted RR or odd risk (OR) or hazard ratio (HR) on the association between adiponectin concentrations and risk of CVD reported in the original articles. HR or OR was assumed to approximate the same measure of RR. The most adjusted one was chose if studies reported estimated effects with more than one degree of adjustment for other risk factors. We pooled data of the included studies using random-effects meta-analysis and reported them as RR with corresponding 95% CI. Heterogeneity across studies was assessed using Q statistic and *I* ^2^ statistics, which is a quantitative measure of inconsistency across studies [20]. Meta-analyses were conducted for the RR of adiponectin concentrations and risk of CVD in type 2 DM patients. We performed sensitivity analyses by omitting 1 study in each time to investigate the inluuence of a single study on the overall risk estimate. We considered substantial heterogeneity exist when *I* ^2^ values were greater than 50%. Subgroup analysis was performed according to study sample size. We also assessed the presence of publication bias using the Begg’s test [21] and the Egger ’s test [22]; the results were considered to indicate publication bias when *p*<0.10.

To enable a consistent approach to analysis in present study, we converted continuous or categorical estimated effect on to a standard scale of effect. We chose the comparison of highest tertile with the lowest tertile of the adiponectin distribution [23]. The scaling methods assume that adiponectin is log normally distributed and that the association with disease risk is log-linear; both these assumptions have empirical support in studies of adiponectin [23]. For a normally distributed variable, the difference between the means of the top and bottom third of the distribution is 2.18 SD units [23]. For two and four groups, scaling factors of 2.18/1.59 and 2.18/2.54 were used, respectively. For normally transformed adiponectin, estimated effects reported per SD increase used a scaling factor of 2.18. All results were estimated by software Stata 11.0 (Stata Corp, College Station, Tex). Statistical tests were 2 sided and used a significance level of *p*<0.05.

## Results

### Description of studies

**Figure 1:**
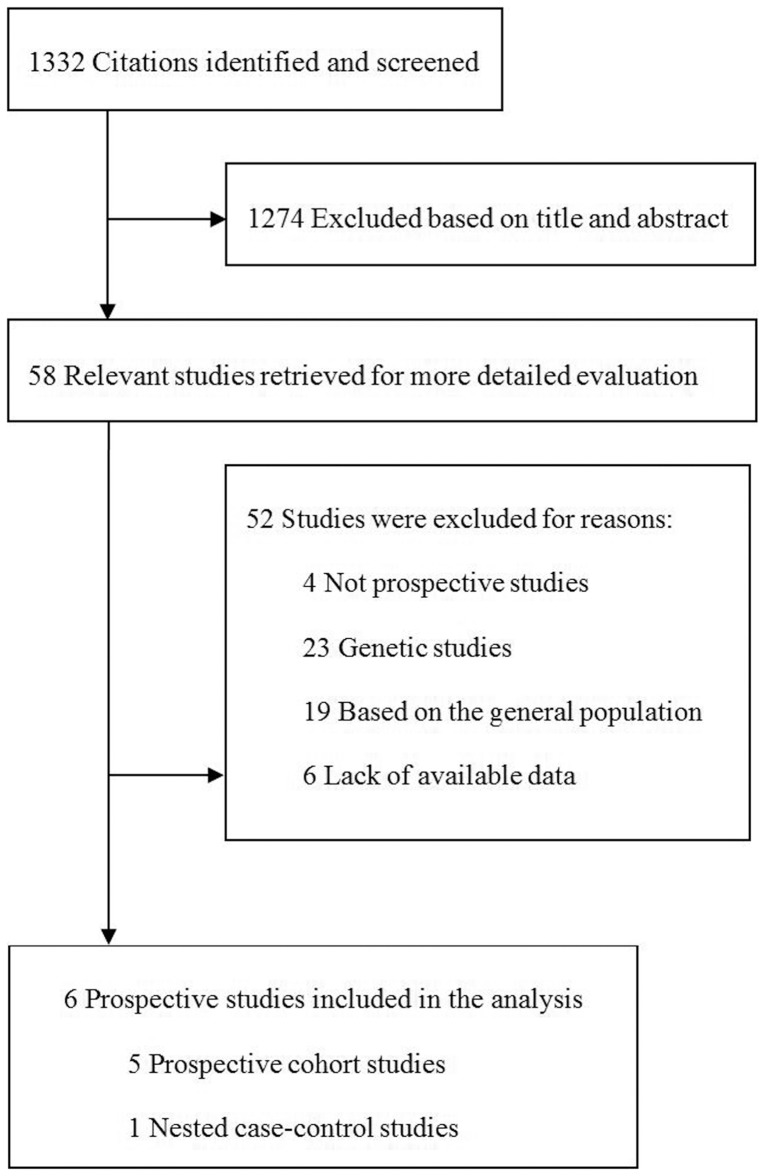
Selection of studies for systematic review of association between adiponectin concentrations and risk of cardiovascular diseases in diabetic patients.

We initially identified 1332 relevant citations. Of these, 1274 citations were excluded after screening the titles and abstracts. After screening the full-test, of these 58 citations, 52 were excluded for the following reasons 1) not prospective studies (n = 4); 2) genetic studies (n = 23); 3) based on the general population (n = 19); 4) lack of available data (n = 6). Finally, the present systematic review and meta-analysis included 5 prospective cohort studies [13]-[17] and 1 nested case-control study, [12] which comprised 2412 diabetic patients (Figure 1). The characteristics of these included studies were shown in Table 1. These studies were published between 2005 and 2013. The sample sizes ranged from 62 [12] to 1038 [17] with a mean of 402 individuals, and the durations of follow-up ranged from 1.3 [16] to 10 [12] years. Two studies were performed in U.S.A., one in German, and three in Asia. Five studies were performed based on type 2 DM patients, and one based on type 1 DM. The proportion of diabetic patients with preexisting CVD in the studies ranged between zero [13], [17] and 100% [16]. These six studies have the number of adjustment variables from 5 to 17. The study quality scores ranged from 5 to 8 (Table 2).

**Table 1:** Characteristics of the identified prospective studies of adiponectin concentrations and risk of CVD.

Study (Authors, year) Reference	Location	Design	Study Population	Patients with preexisting CVD (%)	Number of participants/events	Age (Mean or median, yr)	Type of Adiponectin	Follow-up (yrs)	Outcomes	RRs (95% CI)	Measurements of risks	Adjustment	Quality score^a^
Costacou, 2005	USA	Nested case-control	Patients (type 1 DM)	NA	62/28	34	Total	10	CHD	HR: 0.37 (0.19–0.73)	Per SD	Age, sex, log_10_ AER, HDL and non-HDL cholesterol	8
Schulze, 2005	USA	Prospective cohort	Patients (type 2 DM)	0	745/89	63.1	Total	5	CHD	RR: 0.68 (0.33–1.42)	Quartile	Age, physical activity, family history of MI, history of high BP, history of high blood cholesterol, current aspirin use, smoking, fasting status, duration of diabetes, alcohol, BMI, HDL cholesterol, apoB_100_, triglycerides, CRP, sTNFR2, and fibrinogen	8
Lim, 2008	Korean	Prospective cohort	Patients (type 2 DM)	23.60%	343/38	65	Total	3.5	CVD	HR: 3.03 (1.09–8.41)	Quartile	Age, sex, BMI, creatinine, DM duration, ECG abnormality, CVD history, BP, smoking, lipid status, HbA1c, microalbuminuria and resistin	5
Krzyzanowska, 2009	Japan	Prospective cohort	Patients (type 2 DM)	61%	147/61	63	HMW	1.6	CVD	HR: 0.95 (0.58–1.54)	Per SD	Age, sex, HDL-cholesterol, HbA1c, CRP, GFR, HMW adiponectin, and treatment with biguanides, sulfonyureas, insulin, or statins	5
Hung, 2010	Taiwan	Prospective cohort	Patients (type 2 DM)	100%	77/NA	65	Total	1.3	CVD	RR: 1.18(1.002–1.44)	Equal two groups	Age, sex, waist circumference, MBP, lipid profile, fasting sugar, smoking status, metabolic syndrome, and antihypertensive therapy, statins, and aspirin	6
Schottker, 2013	German	Prospective cohort	Patients (type 2 DM)	0	1038/161	65	Total	8	CVD	HR: 1.48 (1.01–2.21)	Tertile	Age, sex, smoking, physical activity, HbA1c, BP, non-HDL cholesterol, GFR, and NSAIDs	8

a The Newcastle-Ottawa quality assessment scale was chosed for quality assessment, maximum score 9 DM,diabetes mellitus; RR, relative risk; HR, hazard ratio; OR, odds ratio; CI, confidence interval; CVD, cardiovascular disease; CHD, coronary heart disease; SD, standard deviation; HMW, high molecule weigh; NA, not available; HDL, high density lipoprotein; BMI, body mass index; CRP, C-reactive protein; sTNFR2, soluble fractions of tumor necrosis factor-α receptor 2; BP, blood presure; MBP, mean blood pressure; ECG, electrocardiograph; HbA1c, hemoglobin A1c; GFR, glomerular filtration rate; NSAIDs, non-steroidal antiinflammatory drugs; MI, myocardial infarction.

**Table 2:** Assessment of Quality of Studies.

	Selection					Outcome Assessment			
Study (Authors, year)	Representativeness of the Exposed Cohort	Selection of the Nonexposed Cohort	Ascertainment of Exposure	Incident Disease	Comparability	Assessment of Outcome	Length of Follow-up	Adequacy of Follow-up	Score
Costacou, 2005		*	*	*	**	*	*	*	********
Schulze, 2005		*	*	*	**	*	*	*	********
Lim, 2008			*		**	*		*	*****
Krzyzanowska, 2009			*		**	*		*	*****
Hung, 2010		*	*		**	*		*	******
Schottker, 2013		*	*	*	**	*	*	*	********

### Adiponectin concentrations and risk of CVD

**Figure 2:**
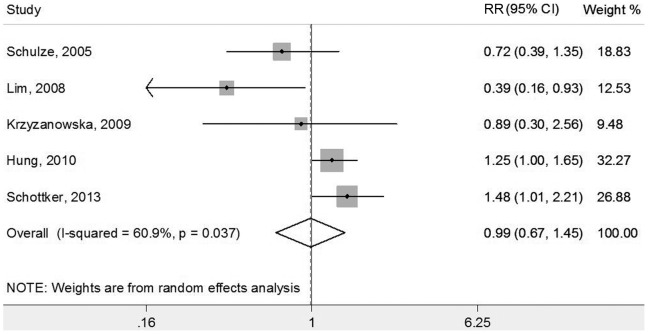
Forest plot showing meta-analysis on the association between adiponectin concentrations and risk of cardiovascular diseases in diabetics.

We evaluate 6 prospective studies in our analysis. One study [12] focus on type 1 DM patients and show an inverse association between adiponectin concentrations and incidence of coronary artery disease (HR = 0.37, 95% CI 0.19–0.73, per 1 SD increase) in multivariable analyses. For the other five studies based on type 2 DM patients, their results show that the relationship was controversial. Lim’s study [14] shows an inverse association (RR = 3.03; 95% CI 1.09–8.41, lowest quartile vs. highest quartile of adiponectin levels), and, quite on the contrary, both Hung [16] and Schottker’s studies [17] report a positive association (RR = 1.18, 95% CI 1.002–1.44, high vs. low group; HR = 1.48, 95% CI 1.01–2.21, top vs. bottom tertile; respectively). Schulze [13] and Krzyzanowska’s studies [15] found that no significant relationship between adiponectin concentrations and incident of CVD (RR = 0.68, 95% CI 0.33–1.42, highest vs. lowest quartile; HR: 0.95, 95% CI 0.58–1.54, per 1 SD increase; respectively). After converting the estimated effect on to the comparison of highest tertile with the lowest tertile of the adiponectin distribution by a relevant scaling method, we conducted a meta-analysis to test the association between adiponectin concentrations and risk of CVD. Result from Lim’s study [14] was converted to the highest quartile vs. lowest quartile of adiponectin concentrations. Five studies (all based on type 2 DM patients) reported association of adiponectin concentrations and risk of CVD, and the pooled RR was 0.99 (95% CI: 0.67–1.45), with significant heterogeneity between studies (*p* = 0.037, *I* ^2^ = 60.9%) (Figure 2).

### Exploration of heterogeneity

Most of the variability across studies is due to true heterogeneity rather than sampling error as tested by an *I* ^2^ value of 60.9%. We perform subgroup analysis and found that there was no significant difference for the pooled estimate between two higher-quality studies [13], [17] (with sample size greater than the median number of 402 cases, patients without preexisting CVD, duration of follow-up greater than the median number of 5 years) and that of other three lower-quality studies [14]-[16] (RR =  1.08, [0.53, 2.17], 0.82, [0.39, 1.73], respectively). The heterogeneity still existed in these two subgroups (*I* ^2^ = 73%, 68.9%, respectively). We then performed a sensitivity analysis by omitting one study at a time and calculating the pooled estimated effects for the remainder of the studies and found that none of the individual studies dramatically affected the overall results with lowest and highest RR with 95% CI were 0.82 (0.48–1.38) and 1.19 (0.92–1.54), respectively. The heterogeneity was partly explained by the Korean study, [14] and when this study was excluded there was small heterogeneity (*I* ^2^ = 26.4%). No evidence of publication bias was found when evaluated by the Begg’s test (*p* = 0.46), or Egger’s test (*p* = 0.15), although these tests were based on only five studies.

## Discussion

Many studies, including several meta-analyses, have been conducted to evaluate the relationship between adiponectin concentrations and risk of CVD in healthy population. But for now there was, to our knowledge, no published meta-analysis to evaluate this association for diabetic patients. The present meta-analysis showed no association was found between adiponectin concentrations and risk of CVD in type 2 diabetic patients. The relationship did not vary by the study sample size, patients without or with preexisting CVD, duration of follow-up, being similar in higher- or lower-quality studies. Our results are consistent with those of two meta-analyses [9], [10] evaluating the association in general population.

Adiponectin has been thought to reduce the risk for future CVD due to one of its function, anti-atherogenic activities [4]. However, recently a meta-analysis based on 16 prospective studies reported that higher concentrations of adiponectin do not reduce risk for CVD [10]. Another meta-analysis included 17 prospective studies also show there was no relationship between adiponectin concentrations and the risk of CHD or CVD [6]. One reason for explaining this phenomenon may be adiponectin isoforms, which dependent the biologic effects of adiponectin. However, the exact biologic effect of each isoform of adiponectin was still controversial [24].

For diabetic patients, the relationship between adiponectin and risk of CVD was mixed. One cohort study [14] based on type 2 DM and one nested case-control study [12] based on type 1 DM concluded that low concentration of adiponectin was a significant risk factor for development of CVD. They explained that adiponectin potentially possesses properties in addition to its anti-atherosclerotic and anti-inflammatory effects. [25], [26].

On the contrary, one cohort study [16] based on type 2 DM with preexisting CVD and another large cohort study [17] based on type 2 DM without preexisting CVD support that higher concentrations of adiponectin were related to higher risk for CVD. In Hung’s study, [16] the reason why type 2 DM patients with preexisting CAD and high adiponectin concentrations were associated with an increased risk of secondary CVD has not been clarified. Schottker et al observed a U-shaped association between adiponectin and risk of CVD in patients with type 2 DM, and indicated that there are differences between diabetic patients and general population in the associations between circulating immune mediators and risk of CVD. [17].

What’s more, other two cohort studies [13], [15] did not found any significant association. Schulze et al [13] found that there was an inverse association between adiponectin and risk of CHD in diabetic men, however, it was no longer statistically significant when adjustment for high density lipoprotein (HDL) cholesterol. They suggest that HDL cholesterol might partly regulate the relationship between adiponectin concentrations and risk of CHD. Krzyzanowska et al [15] support the idea that no significant association was found between adiponectin and risk of CVD in diabetes patients and assumed that although HMW adiponectin owned anti-inflammatory and anti-atherogenic effects, these effects play a more important role in the very early phase of atherogenesis than in manifest macrovascular disease as found in high-risk patients with type 2 DM.

The possible limitations of our meta-analysis must be taken into consideration. First, present meta-analysis is based on published results, more detailed combined analysis of studies using individual participant records could help to characterize dose-response relationship, evaluate associations in particular subgroups, compare directly the magnitude of risk association with adiponectin and CVD, allow more complete adjustment for potential confounding factors. Second, sample size or eligible studies in the present study was relatively small and may have insufficient statistical power to detect the marginal association. However, all the included studies were prospective study design, which reduces, to some extent, selection bias and recall bias compared with retrospective studies. Third, the heterogeneity in our results is substantial because our meta-analysis combines data from studies with different charismatics. However, when one study [14] was excluded, the heterogeneity became small. Fourth, publication bias is a potential concern for any meta-analysis based on published studies. The Begg’s test and Egger’s test suggest no publication bias in this met a-analysis, however, the small number of included studies limits the statistical power in these tests. Finally, we did not included unpublished data and it is also possible that we missed some eligible published studies.

In conclusion, this systematic review and meta-analysis involved 6 prospective studies and indicated that no association was found between adiponectin concentrations and risk of CVD in type 2 diabetic patients. These results should be verified further by large sample size, long duration of follow-up, and well-designed prospective clinical trials.

## Supporting Information

Checklist S1 PRISMA 2009 Checklist.
(DOC)

## References

[1] Maeda K, Okubo K, Shimomura I, Funahashi T, Matsuzawa Y, et al (1996) cDNA cloning and expression of a novel adipose specific collagen-like factor, apM1 (AdiPose Most abundant Gene transcript 1). Biochem Biophys Res Commun 221: 286–289. 8619847 10.1006/bbrc.1996.0587

[2] Goldstein BJ, Scalia RG, Ma XL (2009) Protective vascular and myocardial effects of adiponectin. Nat Clin Pract Cardiovasc Med 6: 27–35. 19029992 10.1038/ncpcardio1398 PMC2658652

[3] Havel PJ (2004) Update on adipocyte hormones: regulation of energy balance and carbohydrate/lipid metabolism. Diabetes 53 Suppl 1S143–151. 14749280 10.2337/diabetes.53.2007.s143

[4] Lihn AS, Pedersen SB, Richelsen B (2005) Adiponectin: action, regulation and association to insulin sensitivity. Obes Rev 6: 13–21. 15655035 10.1111/j.1467-789X.2005.00159.x

[5] Roger VL, Go AS, Lloyd-Jones DM, Adams RJ, Berry JD, et al (2011) Heart disease and stroke statistics—2011 update: a report from the American Heart Association. Circulation 123: e18–e209. 21160056 10.1161/CIR.0b013e3182009701 PMC4418670

[6] Hao G, Li W, Guo R, Yang JG, Wang Y, et al.. (2013) Serum total adiponectin level and the risk of cardiovascular disease in general population: A meta-analysis of 17 prospective studies. Atherosclerosis. 10.1016/j.atherosclerosis.2013.02.018 23489345

[7] Zhang BC, Liu WJ, Che WL, Xu YW (2012) Serum total adiponectin level and risk of cardiovascular disease in Han Chinese populations: a meta-analysis of 17 case-control studies. Clin Endocrinol (Oxf) 77: 370–378. 21995850 10.1111/j.1365-2265.2011.04260.x

[8] Zhang H, Mo X, Hao Y, Huang J, Lu X, et al.. (2012) Adiponectin Levels and Risk of Coronary Heart Disease: A Meta-analysis of Prospective Studies. Am J Med Sci. 10.1097/MAJ.0b013e318262dbef 23123561

[9] Sattar N, Wannamethee G, Sarwar N, Tchernova J, Cherry L, et al (2006) Adiponectin and coronary heart disease: a prospective study and meta-analysis. Circulation 114: 623–629. 16894037 10.1161/CIRCULATIONAHA.106.618918

[10] Kanhai DA, Kranendonk ME, Uiterwaal CS, van der Graaf Y, Kappelle LJ, et al.. (2013) Adiponectin and incident coronary heart disease and stroke. A systematic review and meta-analysis of prospective studies. Obes Rev. 10.1111/obr.12027 23495931

[11] Sarwar N, Gao P, Seshasai SR, Gobin R, Kaptoge S, et al (2010) Diabetes mellitus, fasting blood glucose concentration, and risk of vascular disease: a collaborative meta-analysis of 102 prospective studies. Lancet 375: 2215–2222. 20609967 10.1016/S0140-6736(10)60484-9 PMC2904878

[12] Costacou T, Zgibor JC, Evans RW, Otvos J, Lopes-Virella MF, et al (2005) The prospective association between adiponectin and coronary artery disease among individuals with type 1 diabetes. The Pittsburgh Epidemiology of Diabetes Complications Study. Diabetologia 48: 41–48. 15616802 10.1007/s00125-004-1597-y

[13] Schulze MB, Shai I, Rimm EB, Li T, Rifai N, et al (2005) Adiponectin and future coronary heart disease events among men with type 2 diabetes. Diabetes 54: 534–539. 15677512 10.2337/diabetes.54.2.534

[14] Lim S, Koo BK, Cho SW, Kihara S, Funahashi T, et al (2008) Association of adiponectin and resistin with cardiovascular events in Korean patients with type 2 diabetes: the Korean atherosclerosis study (KAS): a 42-month prospective study. Atherosclerosis 196: 398–404. 17178123 10.1016/j.atherosclerosis.2006.11.017

[15] Krzyzanowska K, Aso Y, Mittermayer F, Inukai T, Brix J, et al (2009) High-molecular-weight adiponectin does not predict cardiovascular events in patients with type 2 diabetes. Transl Res 153: 199–203. 19304279 10.1016/j.trsl.2009.01.009

[16] Hung WC, Wang CP, Lu LF, Yu TH, Chiu CA, et al (2010) Circulating adiponectin level is associated with major adverse cardiovascular events in type 2 diabetic patients with coronary artery disease. Endocr J 57: 793–802. 20818134 10.1507/endocrj.k10e-020

[17] Schottker B, Herder C, Rothenbacher D, Roden M, Kolb H, et al.. (2013) Proinflammatory Cytokines, Adiponectin, and Increased Risk of Primary Cardiovascular Events in Diabetes Patients With or Without Renal Dysfunction: Results from the ESTHER study. Diabetes Care. 10.2337/dc12-1416 PMC3661844 23378623

[18] Liberati A, Altman DG, Tetzlaff J, Mulrow C, Gotzsche PC, et al (2009) The PRISMA statement for reporting systematic reviews and meta-analyses of studies that evaluate health care interventions: explanation and elaboration. J Clin Epidemiol 62: e1–34. 19631507 10.1016/j.jclinepi.2009.06.006

[19] Stang A (2010) Critical evaluation of the Newcastle-Ottawa scale for the assessment of the quality of nonrandomized studies in meta-analyses. Eur J Epidemiol 25: 603–605. 20652370 10.1007/s10654-010-9491-z

[20] Higgins JP, Thompson SG, Deeks JJ, Altman DG (2003) Measuring inconsistency in meta-analyses. BMJ 327: 557–560. 12958120 10.1136/bmj.327.7414.557 PMC192859

[21] Begg CB, Mazumdar M (1994) Operating characteristics of a rank correlation test for publication bias. Biometrics 50: 1088–1101. 7786990

[22] Egger M, Davey Smith G, Schneider M, Minder C (1997) Bias in meta-analysis detected by a simple, graphical test. BMJ 315: 629–634. 9310563 10.1136/bmj.315.7109.629 PMC2127453

[23] Danesh J, Collins R, Appleby P, Peto R (1998) Association of fibrinogen, C-reactive protein, albumin, or leukocyte count with coronary heart disease: meta-analyses of prospective studies. JAMA 279: 1477–1482. 9600484 10.1001/jama.279.18.1477

[24] Kang EH, Lee YJ, Kim TK, Chang CB, Chung JH, et al (2010) Adiponectin is a potential catabolic mediator in osteoarthritis cartilage. Arthritis Res Ther 12: R231. 21194467 10.1186/ar3218 PMC3046544

[25] Kumada M, Kihara S, Ouchi N, Kobayashi H, Okamoto Y, et al (2004) Adiponectin specifically increased tissue inhibitor of metalloproteinase-1 through interleukin-10 expression in human macrophages. Circulation 109: 2046–2049. 15096450 10.1161/01.CIR.0000127953.98131.ED

[26] Yang WS, Lee WJ, Funahashi T, Tanaka S, Matsuzawa Y, et al (2001) Weight reduction increases plasma levels of an adipose-derived anti-inflammatory protein, adiponectin. J Clin Endocrinol Metab 86: 3815–3819. 11502817 10.1210/jcem.86.8.7741

